# A Reliability of Active and Passive Knee Joint Position Sense Assessment Using the Luna EMG Rehabilitation Robot

**DOI:** 10.3390/ijerph192315885

**Published:** 2022-11-29

**Authors:** Łukasz Oleksy, Aleksandra Królikowska, Anna Mika, Paweł Reichert, Monika Kentel, Maciej Kentel, Anna Poświata, Anna Roksela, Dominika Kozak, Katarzyna Bienias, Marcel Smoliński, Artur Stolarczyk, Michał Mikulski

**Affiliations:** 1 Department of Physiotherapy, Faculty of Health Sciences, Jagiellonian University Medical College Krakow, 31-008 Krakow, Poland; 2 Oleksy Medical & Sport Sciences, 37-100 Łańcut, Poland; 3 Ergonomics and Biomedical Monitoring Laboratory, Department of Physiotherapy, Faculty of Health Sciences, Wrocław Medical University, 50-368 Wrocław, Poland; 4 Institute of Clinical Rehabilitation, University of Physical Education in Kraków, 31-571 Kraków, Poland; 5 Department of Trauma Surgery, Clinical Department of Trauma and Hand Surgery, Faculty of Medicine, Wrocław Medical University, 50-368 Wrocław, Poland; 6 eMKaMED Medical Centre, 53-110 Wrocław, Poland; 7 EGZOTech Sp. z o.o., 44-100 Gliwice, Poland; 8 Faculty of Automatic Control, Electronics and Computer Science, Silesian University of Technology, 44-100 Gliwice, Poland; 9 Department of Orthopaedics and Rehabilitation, Medical Faculty, Medical University of Warsaw, 02-091 Warsaw, Poland

**Keywords:** biomedical monitoring, joint position sense, knee, patient monitoring, physiotherapy, proprioception, rehabilitation robotics, reliability, symmetry

## Abstract

Joint position sense (JPS) is the awareness of joint location in space, indicating accuracy and precision of the movement. Therefore, the aim of the present study is to determine the reliability of active and passive JPS assessment regarding the knee joint. This was carried out using the Luna EMG rehabilitation robot. Further analysis assessed whether the examination of only the dominant site is justified and if there are differences between sites. The study comprised 24 healthy male participants aged 24.13 ± 2.82 years, performing sports at a recreational level. Using the Luna EMG rehabilitation robot, JPS tests were performed for the right and left knees during flexion and extension in active and passive mode, in two separate sessions with a 1-week interval. Both knee flexion and extension in active and passive modes demonstrated high reliability (ICC = 0.866–0.982; SEM = 0.63–0.31). The mean JPS angle error did not differ significantly between the right and left lower limbs (*p* < 0.05); however, no between-limb correlation was noted (*r* = 0.21–0.34; *p* > 0.05). The Bland–Altman plots showed that the between-limb bias was minimal, with relatively wide limits of agreement. Therefore, it was concluded that the Luna EMG rehabilitation robot is a reliable tool for active and passive knee JPS assessment. In our study, JPS angle error did not differ significantly between left and right sides; however, the slight asymmetry was observed (visible in broad level of agreement exceeding 5° in Bland–Altman plots), what may suggest that in healthy subjects, e.g., active athletes, proprioception should always be assessed on both sides.

## 1. Introduction

Proprioception of the knee plays a crucial role in maintaining joint stability and coordination during motion [1,2]. Joint position sense (JPS) is awareness of joint location in space, indicating the accuracy and precision of movement [3,4]. Proprioceptive sensory impairment may result from neurological disorders [5,6], chronic soft-tissue diseases [7,8], or neuropathic and orthopaedic conditions [9,10]. Joint position reproduction is the most common method of measuring JPS [11,12,13]; therefore, it is widely used in clinical practice to assess the effectiveness of a rehabilitation programme and identify patients at a higher risk of injury [14]. 

Various methods have been used to assess JPS, including digital inclinometer measurements [1], the image-capture technique [15], inertial sensor-based systems [16], mobile applications [4,17], and some other devices, e.g., customised portable motion capture system with 3D camera [18]. The most commonly used device for knee JPS assessment purposes is an isokinetic dynamometer [17,19]. JPS accuracy is evaluated by so-called angle error, which is the difference between the predefined target joint angle and the reproduced angle measured in active or passive modes [11]. In active mode, a subject is asked to actively move a limb into a predefined ‘target’ position and return to the starting position, and then, to actively move their limb back into this position to replicate the movement. In passive mode, a subject’s limb is moved by a researcher into the desired ‘target’ position and returned to a starting position, then, it is moved again and stopped when the participant reports that the target angle is reached [2].

A crucial issue in JPS evaluation is that the equipment should be easy to use, inexpensive, and give reliable results. The goniometers or inclinometers have been suggested as the best choices, but demonstrated low intra- and inter-rater variability [17,20]. In addition, low reliability of active JPS ICC = 0.36 was reported by Marks et al. [21] in patients with osteoarthritis of the knee, and varied broad range ICCs from 0.07 to 0.89 in young college-aged, healthy adults were observed by Pincivero et al. [22]. On the other hand, the isokinetic dynamometers were reported as highly reliable; but, as was underlined, their use is limited due to high costs and the large size of the equipment [23]. Other studies have reported that motion capture systems or smartphone applications for JPS assessment, may be also valid [4,17,19]. Smith et al. [2] reported good intra-rater reliability for the assessment of JPS using photographs, digital images and replicating knee position using a paper model. However, despite a relatively high number of studies demonstrating the reliability of JPS assessment, the data are often contradictory and inconclusive.

The Luna EMG is a rehabilitation robot designed mainly for therapeutic purposes, with the possibility of using the surface electromyography (sEMG) signal for active-assistive training. The robot also can be applied for evaluation, including assessment of JPS [24]. The evaluation of JPS is clinically the most frequently used measurement of knee proprioceptive capability; however, its reliability and validity remain not fully explained.

In daily functional activities, the limbs on the preferred and non-preferred sides of the body play different roles [25,26]. Due to the fact that previous reliability studies were mainly focused on JPS assessment on the dominant side only, there are a lack of studies in which both sides and the differences between them were analysed. Asymmetry of the musculoskeletal system functioning is a common phenomenon, and the size of asymmetry between the right and left sides may increase in various situations, such as specific athletic training or illness [27]. Little is known about the influence of side dominance on knee JPS; although, in some studies, in active mode, no differences have been found between the right and left legs in healthy young and older subjects [28], while in others, asymmetry in active mode has been noted in young athletes [29]. It seems reasonable to probe whether there are any differences between sides of JPS knee assessment in both passive and active modes. 

Because the reliability of JPS measurements remains not fully explained and contradictory results have been reported in the literature, the novelty of this study contribution is to explain whether:

- The assessment of knee JPS in active and passive mode using a new device is sufficiently reliable for clinical practice;
- The assessment on only one side (most often the dominant side) and considering the results as equal to both limbs is correct, or whether proprioception on the dominant and non-dominant side does not work identically and both limbs should always be tested.

Therefore, the aim of the present study was to determine the reliability of active and passive JPS assessment in the knee joint using the Luna EMG rehabilitation robot. As the second aim, this study is the first to assess whether examining only the dominant limb is justified and if there are any between-limb differences in either active or passive modes.

## 2. Materials and Methods

### 2.1. Study Design

**Figure 1:**
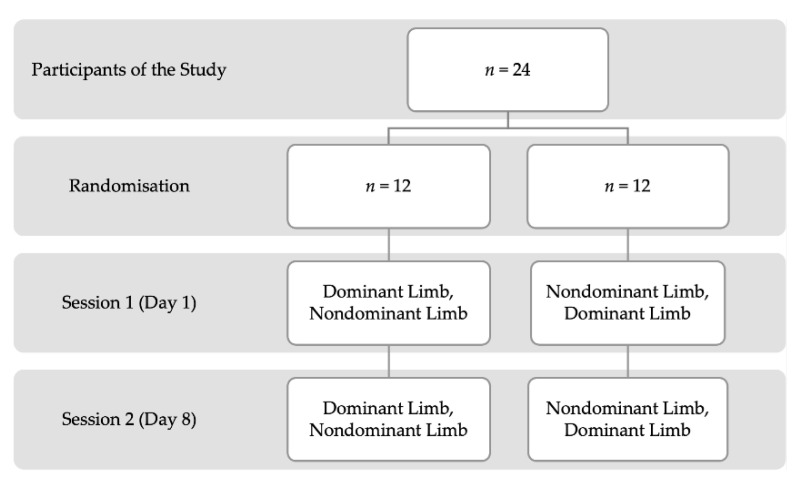
The study procedure.

In the study, a repeated measure, observational design was implemented. The active and passive knee JPS was measured on 2 separate occasions with a 1-week interval between interventions. The trials were carried out in the morning hours at normal room temperature (22–23 °C). The schema of study design is presented on Figure 1. All the measurements of knee JPS were performed in the laboratory by the same rater, who was trained and had good experience with the equipment as well as the test protocol. The participants were asked not to provide changes to their regular training regimens and to avoid vigorous physical activity during the week between both sessions.

### 2.2. Characteristics of Study Participants

Twenty-four male, healthy students aged 18–30 (24.13 ± 2.82) years, volunteered for the study. They all met the inclusion criteria, i.e., not having any pain, injury, and/or disease in the lower limb(s); not having any systematic disease; performing sports at a recreational level; dominant right lower limb. The leg dominance was determined by asking, “If you would kick a ball towards a target, which leg would you use to do so?” [30]. Body mass (kg) and height (cm) were measured prior to testing. Consecutively, body mass index (BMI) was calculated. The participants were recruited through advertising on university social media. Before examination, all participants were informed about the purpose of the study and signed informed consent. The participants were familiarised with all measurement procedures.

### 2.3. Joint Position Sense Measurement Procedure

The JPS measurement was performed using the Luna EMG rehabilitation robot with the Mezos SIT examination and treatment chair (EGZOTech Sp. z o.o., Gliwice, Poland). Luna EMG rehabilitation robot is a medical device used as a tool for rehabilitation and patient evaluation. Exercises and assessments are based on the usage of electromyogram, integrated torque sensor, and position measurements. It allows for clinical evaluations of the muscle motor unit recruitment and activation sequence through surface electromyography, strength by maximal torque measurements, range of motion measurements via passive, assisted and active mode, spasticity, and rigidity measurement during passive movement. The robot was calibrated according to the manufacturer’s instructions before each testing session. During measurements, the participants were dressed in comfortable sports attire and were provided with a black mask to cover their eyes.

**Figure 2:**
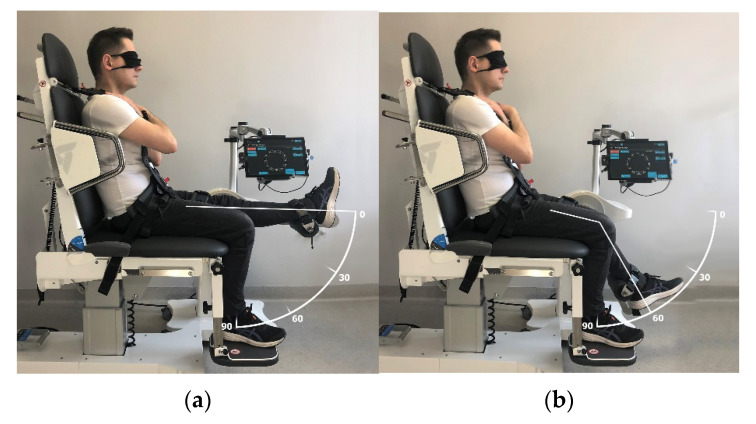
Knee flexion joint position sense (JPS) measurement: (**a**) baseline position; (**b**) target position.

**Figure 3:**
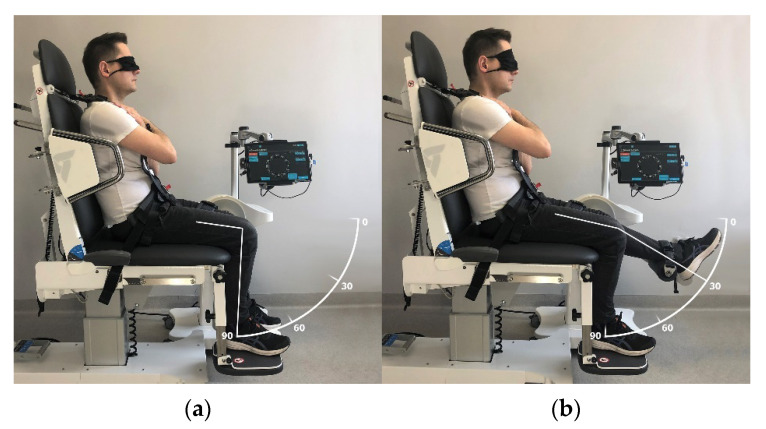
Knee extension joint position sense (JPS) measurement: (**a**) baseline position; (**b**) target position.

The measurements were performed in a seated position with 90° hip flexion and arms crossed over the chest. The trunk and examined limb were stabilised using belts. The JPS measurement consisted of 4 consecutive tests for each lower limb. This is presented in Table 1. The baseline knee joint position was 0° (Figure 2a) when the knee was aimed to flex to a target position of 60°, actively or passively (Figure 2b). Conversely, the baseline position of the knee joint was 90° (Figure 3a) when the knee was to be extended to the target position set at 30° (Figure 3b).

The examination was performed bilaterally; however, the participants were randomly assigned to begin with the right or left lower limb. In athletes and in healthy young participants, two repetitions of each movement were reported as appropriate [31,32]. Two trials were performed for each JPS test and mean was accepted for data analysis.

#### 2.3.1. Joint Position Sense (JPS) Measurement in Active Mode

The examined knee was passively moved to a predetermined target position for active joint position assessment. The knee remained in this position for 5 s so the participant could remember the specific position and was then consecutively and passively moved to baseline position. After maintaining the baseline position for 3 s, the participant actively moved the examined limb to match the target position [31]. The device registered the matching position when the participant verbally informed that he considered the knee to be at the target position.

#### 2.3.2. Joint Position Sense (JPS) Measurement in Passive Mode

The examined knee was first passively moved to a predetermined target position for passive joint position assessment procedure. The knee was locked in this position for 5 s so the participant could remember the specific position. Subsequently, the knee was passively moved to baseline position. After maintaining the baseline position for 3 s, the participant’s knee was passively moved and stopped when he considered that it had reached the target position [31].

### 2.4. Statistical Analysis

Statistical analysis was performed using STATISTICA 13.0 Pl software. The data distribution was evaluated via the Shapiro–Wilk test. Data are mean and SD. For each repetition, the collected parameter was the absolute difference between the target and replicated position, defined as JPS angle error expressed in degrees (°). For further analysis, the mean of the 2 repetitions (mean absolute JPS angle error) was evaluated separately for the right and left limbs. The arithmetic mean and standard deviation (SD) were calculated for participants’ age, body height, body mass, and BMI. The intra-rater reliability of the variables was determined with the intraclass correlation coefficients (ICC) according to Shrout and Fleiss [33]. The interpretation of the ICC agreement was performed according to Koo et al. [34]: below 0.50—poor; between 0.50 and 0.75—moderate; between 0.75 and 0.90—good; above 0.90—high. The variability within each data set was described using the arithmetic mean and standard deviation (SD) with 95% confidence intervals (CI 95%), coefficients of variation (CV) and standard error of measurement (SEM). Because data were not normally distributed, the non-parametric Wilcoxon signed-rank test was used to determine the differences between the right and left knees. In addition, non-parametric Spearman’s correlation coefficient (*r*) was calculated to compare the right and left sides. The bias between 2 means (active vs. passive mode and right vs. left knee) was examined using the Bland–Altman method. Differences were considered statistically significant at the level of *p* < 0.05. The minimal sample calculation was based on Bujang and Baharum’s proposal. These guidelines demonstrated a specific sample size requirement for ICC with previously assumed threshold values for this analysis [35]. In order to determine the repeatability of 2 measurements, analysis was carried out during separate sessions, power analysis indicated that at least 22 subjects were required to obtain a power of 0.8 at alpha = 0.05 with effect size d = 0.8 and a minimum ICC of 0.50. Additionally, 2 participants were added in case of dropouts between the sessions.

## 3. Results

The final sample consisted of 24 male participants aged 24.13 ± 2.82 years, with the body mass being 79.67 ± 11.63 kg, body height totalling 179.75 ± 5.25 cm, and BMI at an average of 24.64 ± 3.32.

### 3.1. Reliability of Active and Passive Modes of Joint Position Sense (JPS)

The highest mean JPS angle error was noted for knee flexion in active mode (6.22–6.31°) and the lowest for knee extension in passive mode (3.68–2.75°). Both knee flexion and extension in active and passive modes demonstrated high reliability (ICC = 0.866–0.982; SEM = 0.63–0.31). The reliability was high on both sides—right and left (Table 2).

### 3.2. Comparison of Right and Left Sides of Joint Position Sense (JPS)

The mean JPS angle error did not differ significantly between the right and left knees or in active or passive modes (*p* < 0.05) (Table 3). Pearson’s correlation between the right and left sides was weak to moderate and non-significant (*r* = 0.21–0.34; *p* > 0.05) (Table 3).

**Figure 4:**
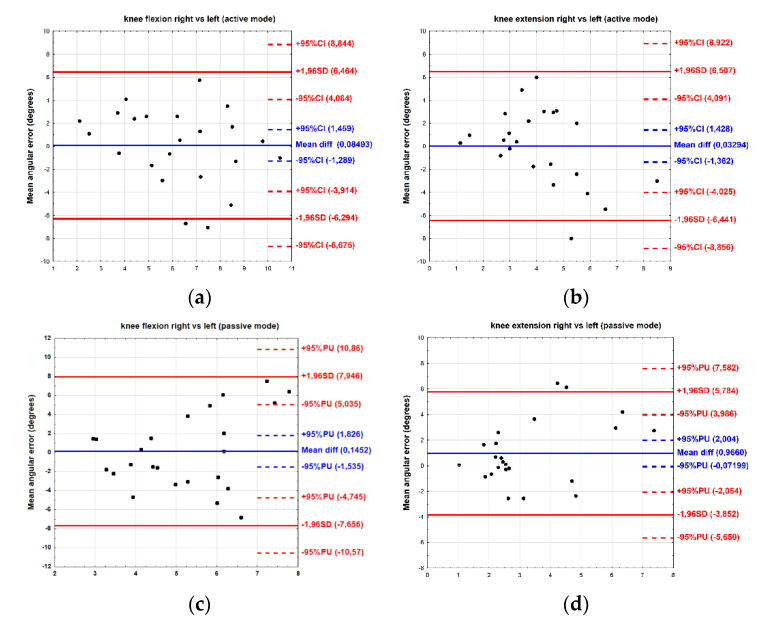
Bland–Altman plot showing agreement between right and left sides for active (**a**,**b**) and passive (**c**,**d**) modes of joint position sense (JPS).

For the active mode, the results of the Bland–Altman plot show that the bias between the right and left sides was minimal. The mean difference for flexion was 0.08° (limit of agreement −6.29 to 6.46°) and for extension was 0.03° (limit of agreement −6.44 to 6.50°). Similarly, for the passive mode, the bias was below 1°. The mean difference for flexion was 0.14° (limit of agreement −7.65 to 5.03°), while for extension, this totalled 0.96° (limit of agreement −3.85 to 3.98°) (Figure 4).

### 3.3. Comparison of Active and Passive Modes of Joint Position Sense (JPS)

**Figure 5:**
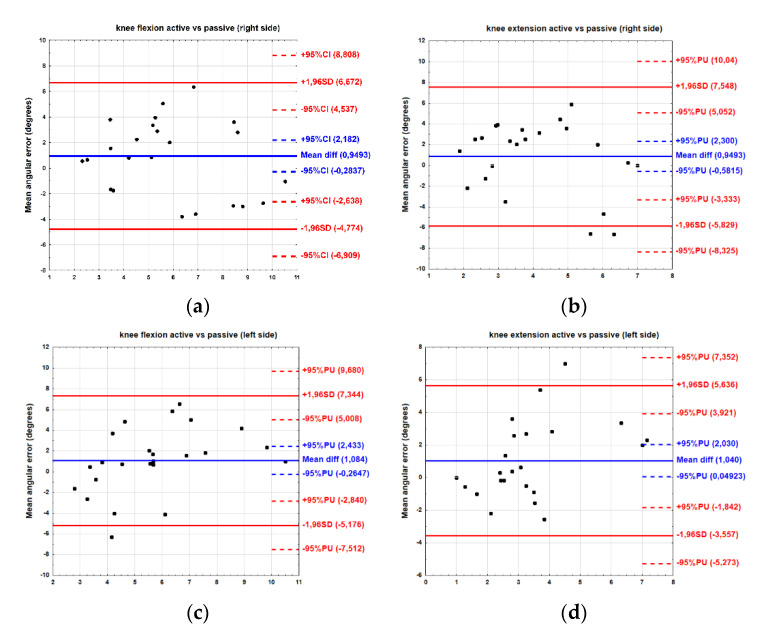
Bland–Altman plot showing agreement between active and passive modes for right (**a**,**b**) and left (**c**,**d**) sides of joint position sense (JPS).

For the right side, the results of the Bland–Altman plot show that the bias between active and passive modes was below 1°. The mean difference for flexion was 0.94° (limit of agreement −4.77 to 6.67°) and for extension, this equalled 0.85° (limit of agreement −5.82 to 7.54°). For left side, the bias was a bit higher, slightly above 1°. The mean difference for flexion was 1.08° (limit of agreement −5.17 to 7.34°) and for extension, this was 1.04° (limit of agreement −3.55 to 5.63°) (Figure 5).

## 4. Discussion

The most important result obtained from this study is that reliability of the knee JPS test performed with the Luna EMG rehabilitation robot was high for both knee flexion and extension in active and passive modes as well on the right as on the left sides. We also observed, that despite the mean JPS angle error not differing significantly between the right and left knees, asymmetry in JPS was noted. However, no between-limb correlation of angular error was observed. Furthermore, the limits of agreement on the Bland–Altman plots were relatively broad-ranged, exceeding 5°, a value previously reported as the minimal clinically significance difference for healthy, non-injured participants [36]. We have also noted that the bias of mean JPS angle error between the passive and active modes was close to 1°for both flexion and extension, indicating a slightly higher JPS angle error in passive mode.

Optimal motor control requires appropriate proprioceptive skills; thus, any deterioration can increase the risk of injury [3]. It has been reported that knee joint position sense assessment may be used for detecting proprioception deficits resulting from injuries, surgical treatment of different knee structures [3], or knee osteoarthritis [19]. To date, JPS testing reliability has been evaluated by different devices [2]. Still, the results reported in the literature on the subject vary among studies due to participants’ age, JPS testing method, tested angle, and the interval between testing sessions [3,15,19]. Therefore, this study is the first in which the reliability of knee JPS measures has been assessed using the Luna EMG rehabilitation robot.

For coaches and health professionals, an important issue in JPS evaluation is that all the equipment should be easy to use, inexpensive, widely available, and give immediate results. Within this context, goniometers or inclinometers have been suggested as the best choices; however, an important drawback is their relatively low intra- and inter-rater variability [17,20]. In contrast, a more advanced method such as video analysis cannot provide JPS values immediately after performing the test and causing the process for obtaining results to be greatly time-consuming [15]. Isokinetic dynamometers—the most sophisticated of tools—are often used especially in laboratory settings, but despite high reliability, their use is limited due to high costs and the large size of the equipment [23]. The Luna EMG rehabilitation robot presented high reliability (ICC = 0.866–0.982; SEM = 0.63–0.31). Moreover, although it is a device dedicated mainly to clinical or laboratory use, it allows to obtain test results immediately after assessment, without any additional data processing. In some studies, it has been reported that sophisticated equipment, i.e., motion capture systems or plain ones such as smartphones, may be similarly valid in JPS assessment [4,17,19]. Our results are consistent with those achieved in the study by Nakashima et al. [4], who showed that the reliability (ICC = 0.930–0.969) of both devices, the iPhone and VICON motion capture, was high and comparable, despite vast differences in design and usage. In their review, Smith et al. [2] reported the reliability of four JPS methods: position replication using a model, image-recorded angulation, electrogoniometry, and a dynamometry/angular motion chair. They concluded that intra-rater reliability was good for the assessment of JPS using photographs, digital images, and replicating knee position using a paper model. It was also good when electrogoniometry was used, and moderate when assessed using dynamometry/angle motion chairs [2]. Therefore, the high reliability observed in our study may suggest that the Luna EMG rehabilitation robot is a valid and useful tool for the purpose of JPS evaluation.

Another important issue in JPS testing is the target joint angle at which the test is performed, but the results from other studies are variable. Selfe et al. [37] examined the effects of knee position on JPS reliability and reported no significant difference in accuracy between measurements obtained at a target angle of 20° compared to 60° (*p* = 0.56). Furthermore, Fatoye et al. [38] reported lower ICC values when JPS was assessed at 10° compared to 25° of knee flexion. Nakashima et al. [4] observed that measurements at both 30° and 60° were reliable for use in clinical evaluation. Nonetheless, they underlined that the results of ICC and correlation coefficients suggested that for 30°, the absolute JPS angle error may have a higher reliability and less bias on Bland–Altman plots than in the case of 60° [4]. They reported that the absolute JPS angle error values, which act as indices of joint position perception, were consistent with those obtained in previous studies [39,40,41], and ranged from 2.82° to 3.59° [4]. In our study, both angles: 30° for knee flexion and 60° for knee extension, demonstrated high reliability (ICC = 0.866–0.982) and low SEM (SEM = 0.63–0.31), indicating that this device is valid in JPS assessment for both flexion and extension in the knee joint. Our data also demonstrated relatively small JPS angle error for both the active and passive modes. Since clinically significant differences of at least 5° have been found in healthy participants [36], our results may suggest the Luna EMG robot is a useful device for clinical JPS assessment. Moreover, Relph et al. [41] reported that the mean difference in absolute JPS angle error values between the ACL-deficient patients and healthy participants was 5.3°.

The mode of evaluation is another significant issue in JPS testing. Most often, active or passive modes are used. Active testing represents the proprioception function needed during volitional positioning of the limb in space requiring sufficient motor control and muscle strength [42]. Conversely, passive testing requires the subject to recognise a pre-determined position reproduced passively, without the need for motor control to actuate the contracting muscles [11]. In our study, the results of Bland–Altman plots allowed to demonstrate that the bias of mean JPS angle error between the passive and active modes was close to 1° for both flexion and extension, indicating slightly higher JPS angle error in passive mode and suggesting, that both modes of measurement are similarly accurate. This maintains agreement with the outcome of the study by Drouin et al. [43], where the isokinetic dynamometer was reported as reliable in assessing both passive and active JPS. It was suggested that patients with knee joint dysfunctions may have muscle weakness and inhibition leading to reduced active repositioning accuracy [11,44]. It has also been suggested that during passive movement, muscles are not active; therefore, fusimotor activity and the sensory feedback from muscle spindles are diminished. Thus, in passive mode, input from cutaneous receptors is the most important in sensory feedback [26]. It therefore appears that a crucial issue in proprioception assessment is to adjust the mode of JPS evaluation to the patient’s condition, despite both modes being reported as similarly accurate.

Some authors have suggested that the time gap between measurements may diminish JPS test reliability because proprioception may be influenced by different factors such as time of day, fatigue, or climatologic conditions [19,44]. A low reliability of active JPS totalling ICC = 0.36 was measured over a 10-day period in patients with osteoarthritis of the knee. Such a level was reported by Marks et al. [21]. Additionally, Pincivero et al. [22] observed ICCs ranging from 0.07 to 0.89 in young college-aged, healthy adults re-tested after 1–2 weeks. In our study, a 1-week interval was implemented between measurements, but all our data indicate that this protocol was valid and reliable in knee JPS assessment.

Another source of bias suggested by some authors is the number of repetitions performed during the JPS test [26]. Most often, joint position reproduction protocols for proprioception assessment use only three to five trials during the test [28,45,46]. This protocol was used in healthy subjects from three age groups (young: 20–35 years old, middle-aged: 40–55 years, and older: 60–75 years) [28], in collegiate athletes [45] and young and older active and sedentary adults [46]. Ashton-Miller et al. [47] suggested that this number may be insufficient for quality proprioceptive testing. Piriyaprasarth et al. [48] reported that in patients with post-stroke proprioceptive deficits, 3 trials resulted in failing the test, so 10 repetitions were just enough for adequate JPS measurement. On the other hand, studies which evaluated athletes or healthy young participants used only two trials [31,32]. However, we also obtained highly reliable data from the two repetitions performed in our study. The reason for this discrepancy between studies may be the subjects’ condition. Therefore, we may suggest that in healthy, non-injured participants, two repetitions may be sufficient enough to obtain valid results. Thus, this may be crucial and of great importance, especially during quick testing for injury risk screening, e.g., in sport.

There are some studies in which the existence of asymmetry between right and left sides in JPS has been indicated in young healthy volunteers [26], but in none of the studies has the reliability of JPS assessment been investigated with regard to both sides. The reported results from the majority of studies on reliability concern only the dominant side [4,38,39,41]. This approach may be vulnerable to errors and, finally, may lead to misleading test interpretation and erroneous diagnosis of proprioception deficits, especially in sport. In non-injured athletes, JPS measurements are mainly aimed at detecting small proprioception deficits in the case of musculoskeletal system monitoring, so the evaluation of one side only may be a potential source of non-detected, increased risk of injury. It has been reported that in daily functional activities, the limbs on the preferred and non-preferred sides of the body play different roles [25,26]. The non-preferred lower limb is usually used in stabilising the body for the preferred lower limb to swing, for instance, when kicking a ball [26]. Therefore, examining only one side in daily practice may lead to errors which, in turn, may increase the risk of injury due to undetected proprioception disorders. There are only a few studies on the influence of side dominance with regard to knee JPS, although in the majority, no differences have been observed between the right and left legs [28,29]. Bakhtiari et al. [29] showed smaller JPS angle errors in the dominant limb, but non-significantly different from the non-dominant limb.

Similarly, no statistically significant differences between the values of maximum torque obtained for the right and left limbs during JPS testing have been reported; therefore, the difference was 3.9% in favour of the non-dominant side [49]. Nonetheless, these results were incongruent with some obtained in other works on knee [28], arm [50], and wrist joint assessment [51]. Han et al. [26] demonstrated a side-general asymmetry in individuals with a strong preference for using their right limbs. This was shown in terms of left side proprioceptive task superiority for both the proximal and distal joints in the upper and lower limbs. Superiority of the left upper limb in proprioception tasks performed by right-handed individuals has been attributed to better utilisation of proprioceptive information by a non-preferred arm/hemisphere system. In our research, the JPS angle errors did not differ significantly between the right and left sides, but the correlations between them were low and non-significant. Moreover, the Bland–Altman plots have shown low bias (below 1° of angular error) between sides, but with a relatively broad level of agreement exceeding 5°. This value was previously reported as a minimal clinically important difference in JPS angle error assessment for healthy, non-injured participants [36]. In addition, the bias between sides was slightly higher in passive than in active mode. This may suggest that the knee proprioception and JPS test in healthy subjects may be different in the dominant side compared to the non-dominant one. All of our participants were right leg dominant; thus, the JPS angle error was a bit higher for the right leg.

One of the primary limitations of the present study is that the measurements were not validated against other devices. Hence, a comparative analysis of JPS testing using the Luna EMG and, for example, isokinetic dynamometers, motion analysis systems or goniometers, will be performed in a future study. Another limitation may be considered the age of the participants being between 18–30 years. As the changes in the central and peripheral nervous system resulting from progressive aging negatively affect proprioception, a reliability study should also be performed among an older population. Furthermore, only healthy participants were recruited; thus, further studies should be carried out among patients with knee dysfunctions. Future research should additionally be focused on assessing JPS reliability with the Luna EMG rehabilitation robot in other joints of the upper and lower limbs, which may allow for better implementation of JPS testing in clinical practice and to determine its usefulness as a tool for evaluation and monitoring. Moreover, two repetitions of each measurement were performed in our study, although the literature suggests more trials, especially during assessment of patients with proprioception impairments, e.g., post-stroke.

## 5. Conclusions

The results from this study allow to demonstrate that the Luna EMG rehabilitation robot is a reliable tool for JPS assessment in both knee flexion and extension, in active and passive modes as well on the right as on the left sides. Despite in our study JPS angle error did not differ significantly between left and right sides, however the slight asymmetry was observed (visible in broad level of agreement exceeding 5° in Bland–Altman plots). It may suggest, that in healthy subjects, e.g., active athletes, proprioception should always be assessed on both sides, because the JPS test performed on only one may be misleading, covering the existing deficits in proprioception being the source of potential injury in the future. Therefore, future research should be focused on the evaluation of JPS in dominant and non-dominant leg in athletes of different kinds of sports.

## Figures and Tables

**Table 1:** Description of joint position sense (JPS) assessment procedure.

Knee Movement	Flexion	Extension	Flexion	Extension
Mode	Active	Active	Passive	Passive
Consecutive tests	Test 1	Test 2	Test 3	Test 4
Baseline knee joint position (°)	0	90	0	90
Target knee joint position (°)	60	30	60	30
Time duration for target position (s)	5	5	5	5
Time duration for baseline position (s)	3	3	3	3
Number of repetitions	2	2	2	2

**Table 2:** Reliability results for active and passive joint position sense (JPS) tests.

		Right Side				Left Side			
	Outcome Measure	ICC (Cl 95%)	Mean ± SD (CI 95%)	CV	SEM	ICC	Mean ± SD (CI 95%)	CV	SEM
1st	Flexion active (°)	0.866 (0.765–0.951)	6.29 ± 2.40 (5.28–7.31)	38.16	0.49	0.945 (0.897–0.980)	6.28 ± 3.12 (4.96–7.60)	49.79	0.63
2nd			6.31 ± 2.34 (5.32–7.29)	37.12	0.47		6.22 ± 3.06 (4.93–7.52)	49.26	0.62
1st	Extension active (°)	0.922 (0.801–0.962)	4.43 ± 1.98 (3.56–5.27)	44.82	0.40	0.962 (0.949–0.987)	3.79 ± 2.40 (2.77–4.80)	63.40	0.49
2nd			4.04 ± 1.77 (3.29–4.79)	43.79	0.36		4.01 ± 2.85 (2.80–5.21)	71.03	0.58
1st	Flexion passive (°)	0.982 (0.959–0.992)	5.34 ± 2.98 (4.06–6.61)	55.87	0.60	0.949 (0.927–0.973)	5.37 ± 1.95 (4.55–6.20)	36.35	0.39
2nd			5.28 ± 2.81 (4.10–6.47)	53.15	0.57		5.14 ± 2.00 (4.29–5.98)	39.08	0.41
1st	Extension passive (°)	0.945 (0.877–0.975)	3.57 ± 2.65 (2.45–4.69)	74.12	0.54	0.980 (0.952–0.991)	2.75 ± 1.54 (2.09–3.40)	56.26	0.31
2nd			3.68 ± 2.45 (2.64–4.71)	66.60	0.50		2.71 ± 1.61 (2.03–3.39)	59.25	0.32

SD—standard deviation; ICC—intraclass correlation coefficients; CI 95%—95% confidence intervals; CV—coefficients of variation; SEM—standard error of measurement.

**Table 3:** Agreement between right and left side mean joint position sense (JPS) angle error.

Outcome Measure	*r*		Mean ± SD			
	1st Session	2nd Session	1st Session R/L	*p*	2nd Session R/L	*p*
Flexion active (°)	0.29	0.29	6.62 ± 2.40/6.28 ± 3.12	n.s.	6.31 ± 2.34/6.22 ± 3.06	n.s.
Extension active (°)	0.30	0.30	4.43 ± 1.98/3.79 ± 2.40	n.s.	4.04 ± 1.77/4.01 ± 2.85	n.s.
Flexion passive (°)	0.30	0.34	5.34 ± 2.98/5.37 ± 1.95	n.s.	5.28 ± 2.81/5.14 ± 2.00	n.s.
Extension passive (°)	0.21	0.32	3.57 ± 2.65/2.75 ± 1.54	n.s.	3.68 ± 2.45/2.71 ± 1.61	n.s.

SD—standard deviation; *p*—*p* value; *r*—Spearman’s correlation coefficient.

## Data Availability

The data presented in this study are available upon reasonable request from the corresponding author.
